# Patterned Electrode Assisted One‐Step Fabrication of Biomimetic Morphing Hydrogels with Sophisticated Anisotropic Structures

**DOI:** 10.1002/advs.202102353

**Published:** 2021-10-27

**Authors:** Qing Li Zhu, Chen Fei Dai, Daniel Wagner, Olena Khoruzhenko, Wei Hong, Josef Breu, Qiang Zheng, Zi Liang Wu

**Affiliations:** ^1^ Ministry of Education Key Laboratory of Macromolecular Synthesis and Functionalization Department of Polymer Science and Engineering Zhejiang University Hangzhou 310027 China; ^2^ Bavarian Polymer Institute and Department of Chemistry University of Bayreuth Universitätsstrasse 30 Bayreuth 95440 Germany; ^3^ Department of Mechanics and Aerospace Engineering Southern University of Science and Technology Shenzhen 518055 China

**Keywords:** biomimetic structures, electrical orientations, hydrogels, morphing materials, soft actuators

## Abstract

Anisotropic structures are ubiquitous in nature, affording fascinating morphing behaviors. Biomimetic morphing materials can be developed by spatially controlling the orientations of molecules or nanofillers that produce anisotropic responses and internal stresses under external stimuli. However, it remains a serious challenge to fabricate materials with sophisticated anisotropic architectures. Here, a facile strategy to fabricate morphing hydrogels with elaborately ordered structures of nanosheets, which are oriented under distributed electric field and immobilized by polymerization to form a poly(*N*‐isopropylacrylamide) matrix, is proposed. Diverse sophisticated anisotropic structures are obtained by engineering the electric field through the patterns and relative locations of the electrodes. Upon heating, the monolithic hydrogels with through‐thickness and/or in‐plane gradients in orientation of the nanosheets deform into various three‐dimensional configurations. After incorporating gold nanoparticles, the hydrogels become photoresponsive and capable of programmable motions, for example, dynamic twisting and flipping under spatiotemporal stimuli. Such a strategy of using patterned electrodes to generate distributed electric field should be applicable to systems of liquid crystals or charged particles/molecules to direct orientation or electrophoresis and form functional structures. The biomimetically architectured hydrogels would be ideal materials to develop artificial muscles, soft actuators/robots, and biomedical devices with versatile applications.

## Graphical abstract

Biomimetic morphing hydrogels with sophisticated anisotropic structures are developed by using a distributed electric field to orient the nanosheets that are then immobilized in a thermoresponsive matrix. The electric field can be facilely tuned by structured electrodes with tailored patterns and positions. The resultant hydrogels with specific gradient of orientations exhibit programmed deformations and motions upon spatiotemporal stimulation.

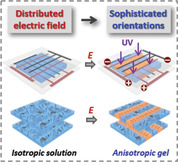

## 1 Introduction

**Figure 1:**
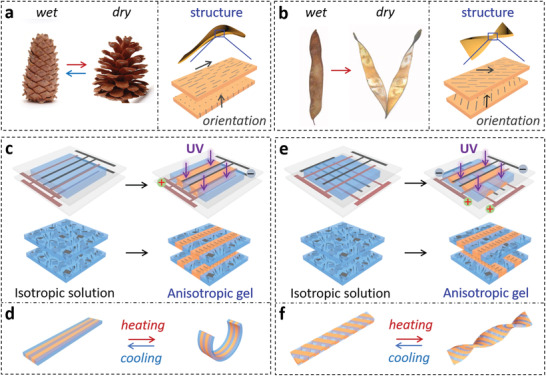
Anisotropic structures and morphing behaviors in natural and artificial systems. a,b) Morphing behaviors of plant organs with different alignments of cellulose fibrils at different regions: a) Pine cones reversibly open and close their scales at low and high humid conditions; b) *Bauhinia* pod opens its valves by deformation into twisted helices upon desiccation. a,b) Reproduced with permission.^[^ ^3^ ^]^ Copyright 2011, American Association for the Advancement of Science. c,e) Schematic for the fabrication and d,f) morphing behaviors of hydrogels with complex ordered structures by using patterned electrodes for electrical orientations of the nanosheets. Anisotropic structures of the hydrogel sheets in (c,e) are produced by electrical orientation of nanosheets under a distributed electric field that is controlled by the pattern and position of the electrodes. The hydrogels are depicted as the upper and the bottom layers to show the alignments of nanosheets near the structured electrodes. Grey and red brown stripes represent the electrodes with positive or negative polarities, and grey sheets represent nanosheets; blue and orange regions correspond to isotropic and anisotropic solution/hydrogel, respectively.

Living organisms invoke complex cellular and molecular processes to form well‐defined, sophisticated anisotropic structures, affording superior mechanical properties or fascinating morphing and motion capacities.^[^ ^1^, ^2^, ^3^, ^4^, ^5^ ^]^ For example, pine cones and the *Bauhinia* pods possess layered microstructures with specific orientations of cellulose fibrils, enabling the pine cone to close/open its scales upon humidity changes and the seedpod to open its halves by twisting deformation upon dehydration (**Figure** **1**a,b). These natural systems have been the inspiring sources for engineering shape‐morphing materials and devices.^[^ ^6^, ^7^, ^8^, ^9^, ^10^ ^]^ With the programmable structures and diverse responses, these materials exhibit abundant morphing behaviors and complex configurations with promising applications as artificial muscles,^[^ ^11^, ^12^, ^13^ ^]^ optical devices,^[^ ^14^, ^15^, ^16^ ^]^ and soft actuators/robots.^[^ ^17^, ^18^, ^19^, ^20^, ^21^, ^22^, ^23^, ^24^ ^]^ Shape‐morphing of materials is generally driven by the nonuniform internal stresses caused by differential contraction/expansion of different parts,^[^ ^25^, ^26^, ^27^, ^28^, ^29^, ^30^, ^31^, ^32^ ^]^ which is usually a result of controlled spatial distribution of components or the alignment of molecules/nanofillers within the soft matrices.^[^ ^8^, ^28^, ^29^, ^30^ ^]^ An extensively studied artificial material is the liquid crystalline elastomers (LCEs). Shape morphing of LCEs can be programmed by locally controlling the alignments of the mesogens using rubbed substrates or photo‐alignment technique.^[^ ^31^, ^32^, ^33^, ^34^, ^35^ ^]^ However, the samples are usually limited in the form of thin films with the thickness in micrometer level, and the procedure for the compartmentalized alignments is complex and time‐consuming.

Hydrogels with multiple responses to stimuli and similarities to biotissues are desirable to design soft actuators and robots with morphing capacity. In recent years, several advanced techniques including photolithography,^[^ ^26^, ^27^, ^28^, ^30^ ^]^ foaming technique,^[^ ^36^ ^]^ 3D/4D printing,^[^ ^25^, ^37^, ^38^, ^39^ ^]^ and magnetic/electric field‐directed assembly^[^ ^40^, ^41^ ^]^ are applied for the fabrication of hydrogels with through‐thickness and/or in‐plane gradients. It has been realized that materials with through‐thickness gradients exhibit bending or folding deformations, while such with in‐plane gradients show buckling deformation with the feature of bistability. However, compared to anisotropic structures in natural systems, the artificial morphing materials usually have gradient structures with amorphous components that exhibit different responses to stimuli. The challenge lies in the fabrication of soft materials with sophisticated yet programmable anisotropic structures, although monodomain hydrogels have been fabricated by using electric/magnetic field^[^ ^42^, ^43^, ^44^ ^]^ or mechanical shear^[^ ^45^, ^46^ ^]^ to orient molecules or nanoparticles before or during the gelation process. In recent years, Studart and coworkers have used multi‐step magnetic field‐induced orientation and site‐specific polymerization to develop a monolithic hydrogel with multiple units having different alignments of nanosheets (NSs) in the upper and the bottom layers.^[^ ^8^ ^]^ The integrated poly(*N*‐isopropylacrylamide) (PNIPAm) hydrogel with through‐thickness gradients exhibited complex shape change and formed a sophisticated configuration upon heating. Li et al. prepared anisotropic hydrogels with through‐thickness gradients by multi‐step electrophoresis of MXene NSs under a direct‐current electric field.^[^ ^42^ ^]^ The resultant hydrogels showed site‐specific bending deformations upon light irradiation. However, the stepwise fabrication procedure is complicated and time‐consuming, and the integrity of the materials is impaired due to the new interfaces created by separate reactions. It is highly desired to develop a facile strategy to fabricate anisotropic hydrogels with intricate in‐plane and/or through‐thickness gradients by spatially controlling the alignments of molecules or nanoparticles toward programmable deformations and motions.

Herein, we propose and demonstrate a versatile strategy to develop biomimetic morphing hydrogels with sophisticated anisotropic structures by using patterned electrodes to generate a distributed electric field for electrical orientation of NSs. Transparent and conductive indium tin oxide (ITO) is patterned on the glass substrate and serves as the electrode that creates an intricate yet controllable electric field, in which the highly charged NSs form complex ordered structures that are immobilized in the hydrogel by polymerization (Figure 1c,e). Various sophisticated anisotropic structures can be obtained by one‐step electrical orientation and polymerization using electrodes with controlled patterns and positions. The resultant hydrogels with localized through‐thickness or in‐plane gradients in orientation of the NSs show programmable deformations upon external stimuli (Figure 1d,f). Monolithic hydrogels consisting of multiple units with distinct gradient structures exhibit sophisticated shape changes to form various 3D configurations on demand. After incorporating gold nanoparticles (AuNPs) as the photothermal transducers, the hydrogels exhibit dynamic deformations and motions under spatiotemporal light stimulations—a concept, that is compelling for the design of soft actuators and robots.

## 2 Results and Discussion

**Figure 2:**
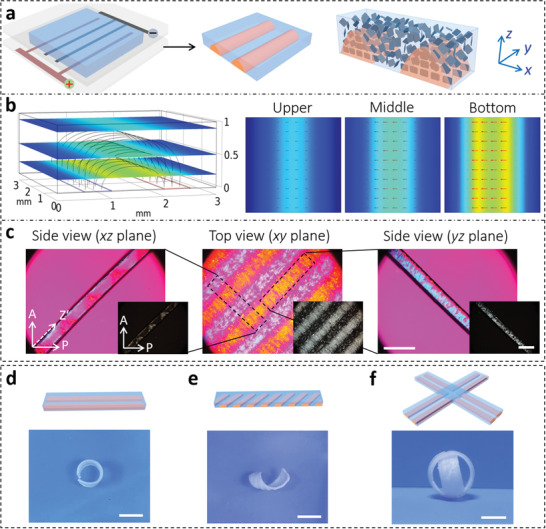
a) Schematic for fabrication of hydrogel with through‐thickness gradient by using electrodes patterned on one substrate for electric orientation of nanosheets. The other substrate of the reaction cell has no electrodes. b) Simulated electric field within 1/2 unit cell of the gel when voltage is applied through the patterned electrodes. The field‐strength distributions on three sections are plotted, together with the in‐plane components of the electric field shown by red arrows. c) POM images of the hydrogel observed from top and side directions. A: analyzer; P: polarizer; Z′: slow axis of the 530 nm tint plate. Scale bar, 3 mm. d) Roll and e) helix configurations of the patterned gels upon heating. f) Design of a four‐arm gripper by tailoring the pattern of electrodes. Scale bar, 10 mm. Gel thickness is 1 mm.

The melt synthesized crystals of sodium fluorohectorite [Na_0.5_]^inter^[Li_0.5_Mg_2.5_]^oct^[Si_4_]^tetr^O_10_F_2_ spontaneously delaminate into singular NSs by osmotic swelling in water.^[^ ^47^, ^48^, ^49^ ^]^ The NSs have high aspect ratio of ≈20 000 and high charge density of 1.1 nm^−2^. In addition, the NSs have anisotropic mechanical properties; they are stiff in the in‐plane direction with the modulus of 150 GPa and flexible in the cross‐plane direction.^[^ ^50^ ^]^ An aqueous suspension of the NSs exhibits a nematic phase at a content as low as 0.3 wt% (Figure S1, Supporting Information). The multi‐domain mesophase suspension is easily oriented into monodomain under uniform electric field due to electrical orientation of the NSs.^[^ ^18^, ^51^ ^]^ It is rational to expect that complex ordered structures of NSs can be produced by a distributed electric field, which can be generated by electrodes of specific patterns and arrangements.^[^ ^52^ ^]^ **Figure** **2**a shows the fabrication of anisotropic hydrogels with through‐thickness gradients by using staggered pectinate electrodes that are plated on one substrate of the reaction cell (the other substrate of the reaction cell has no electrodes). The width and spacing of the strip electrodes are 0.5 and 1.0 mm, respectively. For the electrical orientation of NSs, the strength of the alternating‐current (AC) electric field is set as 4 V mm^−1^ and the frequency as 10 kHz. After applying the electric field for 60 min, the NSs align along the electric field in the strong‐field regions between the electrodes of opposite polarities, whereas the nematic solution in the low‐field regions between the electrodes of same polarities still maintains the multi‐domain state (i.e., macroscopically isotropic). This is confirmed by the computed electric field distribution within a unit cell when voltage is applied through the patterned electrodes (Figure 2b). The color indicates the relative field strength and the curves represent the field lines. The field‐strength distributions on the three sections are also plotted, together with the in‐plane components of the electric field shown by red arrows. The electric field is stronger in the bottom layer between the electrodes with opposite polarities, and weaker at the upper layer. The NSs align along the electric‐field direction, and then this structure is immobilized in the hydrogel after polymerizing the precursor solution of *N*‐isopropylacrylamide (NIPAm). The alignments of NSs in the hydrogel are confirmed by the alternating yellow and pink birefringent strips under a polarizing optical microscope (POM) (Figure 2c; Figure S2, Supporting Information). The hydrogel also shows a through‐thickness gradient due to the asymmetrical arrangement of the electrodes; the layer of the gel (thickness: ≈0.3 mm) close to the substrate without electrodes is amorphous, as confirmed by cross‐sectional observation of the gel. Worth noting is that the AC electric field results in orientation of the NSs rather than migration of molecules or nanoparticles. Therefore, the resultant hydrogel only has a gradient in the orientations of NSs, and the composition is homogeneous throughout the hydrogel (Figure S3, Supporting Information).

The ordered structure provokes anisotropic deformation of the gel, when the temperature is above the lower critical solution temperature (LCST) of PNIPAm. However, the response of the amorphous gel is isotropic. Upon heating, strong internal stress is generated within the patterned hydrogel due to the mismatched deformation, resulting in the gel strips rolling into a tubular or helical shape, with the isotropic layer inside (Figure 2d,e). By varying the pattern of the electrodes, hydrogels with various through‐thickness gradients can be developed, which exhibit programmable deformations to form distinct configurations under external stimuli. As shown in Figure 2f, the cross‐shaped hydrogel deforms and behaves as a four‐arm gripper upon heating.

**Figure 3:**
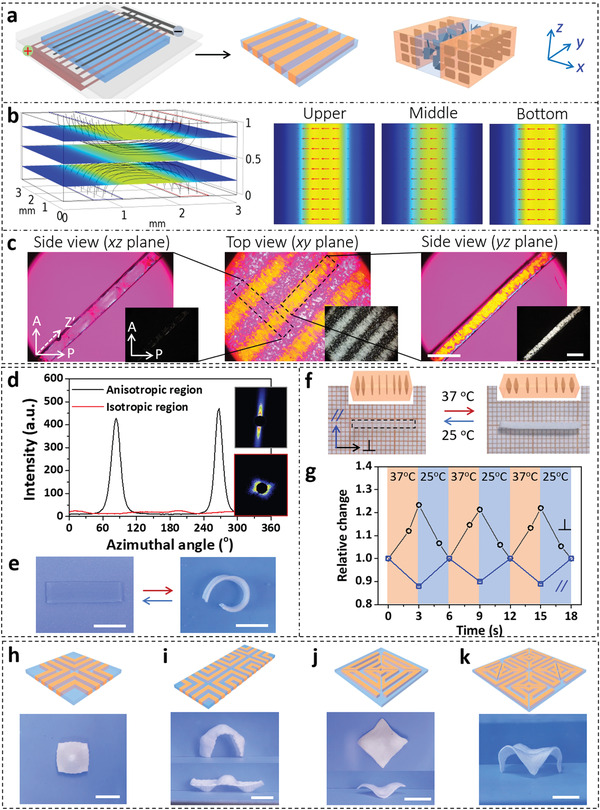
a) Fabrication of in‐plane gradient hydrogel by using electrodes plated on two substrates for electric orientation. b) Simulated electric field of 1/2 unit cell when voltage is applied through the patterned electrodes. c) POM images of the hydrogel observed from top and side directions. Scale bar, 3 mm. d) SAXS patterns and intensity‐azimuth plots of the anisotropic and isotropic regions. Reversible deformation of the e) patterned gel and the f) anisotropic strip, as well as g) the dimensional change. Hydrogels with various in‐plane gradients that deformed into h) dome‐, i) arch/sailboat‐, j) saddle‐, and k) desk‐like configurations upon heating. Scale bar, 1 cm.

Anisotropic hydrogels with in‐plane gradients can be developed by using a pair of patterned electrodes plated on both substrates of the reaction cell. The electrodes with the same pattern are assembled face‐to‐face to eliminate the through‐thickness gradient, yet an in‐plane gradient is generated due to the different alignments of NSs between the electrodes of opposite and same polarities. A representative example is shown in **Figure** **3**a; relatively uniform electric fields in the regions between the electrodes of opposite polarities are confirmed by numerical simulation (Figure 3b). The alternating anisotropic and isotropic regions are confirmed by POM observation and small angle X‐ray scattering (SAXS) measurements (Figure 3c,d). According to the intensity‐azimuth plot, the orientation degree of NSs in the anisotropic region is 0.9. The highly ordered structure endows the hydrogel with an anisotropic response to temperature, driving the flat gel to form an arc (Figure 3e). The response of anisotropic and isotropic gel stripes is examined by slicing the patterned gel and immersing it into hot water. The anisotropic stripe expands by 1.23 in the direction perpendicular to the alignment of NSs, and contracts to 0.88 in the parallel direction (Figure 3f,g). This anisotripic deformation is fast and reversible when the conditions (37 and 25 °C) of the gel are cyclically switched. In contrast, there is no dimensional change within a short time (<30 s) for the macroscopically isotropic gel stripe in hot water. The mismatched dimensional change in the patterned gel is beneficial for the programmable deformations. For example, a square hydrogel with programmed arrangements of NSs is fabricated by using a pair of patterned electrodes, which buckles upward or downward to from a dome upon heating (Figure 3h). After integrating three units, the composite hydrogel deforms into arch‐ or sailboat‐like configurations via selective control of the buckling direction of each unit (Figure 3i), which can be completed by localized heating or pre‐swelling to build a transient through‐thickness gradient in each unit.^[^ ^53^, ^54^, ^55^ ^]^ By controlling the alignments of NSs, the square hydrogel can also deforms into a saddle‐like configuration (Figure 3j). The alignment of NSs is observed by POM in Figure S4, Supporting Information. After integrating four units, the composite deforms into a desk‐like configuration with alternating Gaussian curvatures,^[^ ^3^, ^56^ ^]^ consisting of four saddle shapes (Figure 3k).

The shape change of the composite hydrogel is very fast and completes within 3 s after being transferred into 37 °C water bath (Movie S1, Supporting Information). The quick deformation is result of the temperature‐induced variation of permittivity of water in dehydrated PNIPAm, which consequently changes the electrostatic repulsion between the highly charged NSs.^[^ ^18^, ^44^ ^]^ Owing to the reversibility of this physical process, no fatigue is observed in the temperature‐mediated deformation of the hydrogel for at least ten cycles. The fast deformation of the hydrogel and the high efficiency of electrical orientation are closely related to the high charge density and large aspect ratio of the NSs. In contrast, the graphene oxide (GO) under the same electric field has a relatively low degree of orientation, and the resultant hydrogel only shows slow and weak anisotropic deformation, likely due to the much lower charge density of GO when compared to the fluorohectorite NSs.

**Figure 4:**
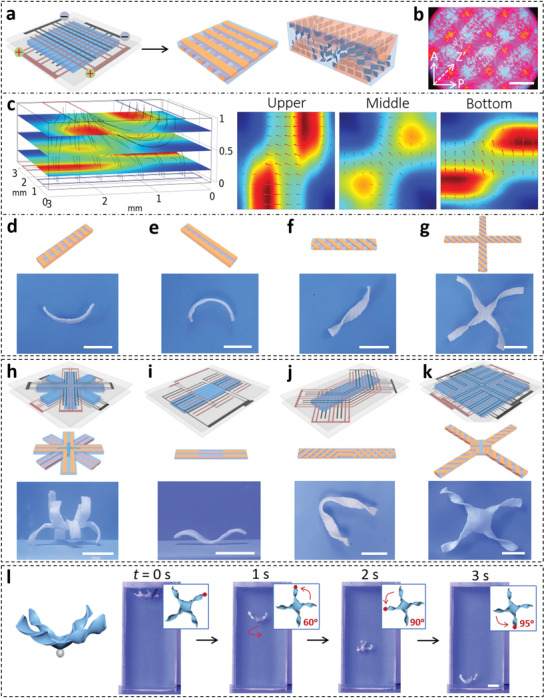
a) Fabrication of hydrogel with complex ordered structures by using orthogonally positioned electrodes patterned on two substrates. b) POM image of the gel viewed from the top. Scale bar, 3 mm. c) Simulated electric field of 1/4 unit cell when voltage is applied through the patterned electrodes. d–g) Various configurations of gel samples cut from the bulk patterned gel at different directions. Scale bar, 1 cm. h–k) Sophisticated deformations of hydrogels prepared by tailoring the pattern and position of electrodes patterned on two substrates. Scale bar, 1 cm. l) Spinning of the jellyfish‐like hydrogel with chiral tentacles during the free fall in 37 °C water bath. Insets illustrate the rotation direction and degree. Scale bar, 2 cm.

Besides patterning, the relative position of the electrodes also influences the gradient structures and corresponding programmed deformations of the anisotropic hydrogels. When two pectinate electrodes with identical pattern are orthogonally positioned on the two substrates, the alignments of NSs in the upper and bottom layers of the gel are mainly determined by the nearby electrodes, resulting in a bilayer‐like hydrogel with stacked anisotropic structures,^[^ ^57^ ^]^ as confirmed by POM observations (**Figure** **4**a,b; Figure S5, Supporting Information). Such complex anisotropic structures are formed by electrical orientations of NSs under the sophisticated electric field, as revealed clearly by the simulation result (Figure 4c). Various gel strips are cut from the bulk hydrogel in different directions that deform into distinct configurations. The gel strips cut along the directions of the bottom and upper electrode bend upward and downward upon heating, respectively (Figure 4d,e). The gel strips cut at 45° and −45° relative to the bottom electrode deform into left‐ and right‐handed twisted helices, respectively (Figure 4f,g). This is because the expansion/contraction mismatch in the upper and bottom layers produces internal stresses along the stripes, and the orthogonal internal stress results in localized saddle‐like curvature and global twisted helix of the gel.^[^ ^57^ ^]^ This mechanism is similar to that of twisting deformation in pod valves (Figure 1b).^[^ ^3^ ^]^

Complex morphing and sophisticated 3D configurations can be programmed in the hydrogels by tailoring the through‐thickness and/or in‐plane gradients with the assistance of patterned electrode‐directed orientations of NSs. As shown in Figure 4h, an eight‐arm gel is fabricated by using two sets of cross‐arrangement electrodes patterned on both substrates with a rotation of 45°, which results in alternating through‐thickness gradients in the arms of the gel. Upon heating, the gel deforms into a blooming‐flower‐like configuration, with four arms bending upward and the other four downward. A serial stack of three units with specific through‐thickness gradient and thus directional bending produces a wave‐like configuration (Figure 4i). Twisting and buckling can also be integrated into one gel strip by compartmentalized through‐thickness and in‐plane gradients. As shown in Figure 4j, the left and right parts twist into helices of different chirality, whereas the middle part buckles without twisting. Even more complex structures can be encoded into one composite. As shown in Figure 4k, four arms, capable of twisting, are integrated at the four corners of the square gel that has the capacity to buckle out of plane. This composite deforms into a jellyfish‐like configuration with twisted tentacles. The chiral tentacles produce asymmetrical hydrodynamics when the gel is moving under water. To confirm this effect, a small glass ball is glued to the central square gel, which is placed in 37 °C water to freely sink. As shown in Figure 4l and Movie S2, Supporting Information, spinning by 245° is observed when the gel falls from a height of 18 cm, indicating a torque produced by the chiral tentacles. In contrast, the hydrogel falls without spinning in 25 °C water when the chiral structures are absent (Figure S6, Movie S3, Supporting Information).

We should note that the design and selection of the electrode patterns are based on the previous understanding of the deformation modes and corresponding internal‐structure features of the hydrogels. It is well‐known that a through‐thickness gradient structure results in bending deformation of a slender hydrogel strip, an in‐plane gradient structure leads to buckling deformation with the nature of bistability, and a bilayer structure of the gel strip with orthogonal internal stresses in the upper and bottom layers (±45° relative to the long axis) forms a twisted helix upon stimulations.^[^ ^57^, ^58^, ^59^ ^]^ An integration of multiple building blocks with specific gradient structures affords complex configuration of the composite hydrogel under external stimuli.^[^ ^8^, ^60^ ^]^ Although most morphing hydrogels are designed with gradients in components that have different responses to stimuli, gradients in the orientations of NSs have similar functions for the programmed deformations of the hydrogels. As demonstrated above, the sophisticated orientations of NSs can be programmed by the patterns and relative positions of the electrodes. However, it remains a big challenge to simulate the shape change and the final configurations of the anisotropic hydrogels, because of the much more sophisticated orientations of NSs which are not uniform throughout the thickness of the gel sheet. Furthermore, the boundaries between adjacent domains with different alignments of NSs are not very sharp, which is different from the systems of LCEs.^[^ ^34^, ^61^ ^]^ Interesting future studies will put more effort into simulating the morphing behaviors of these hydrogels.

**Figure 5:**
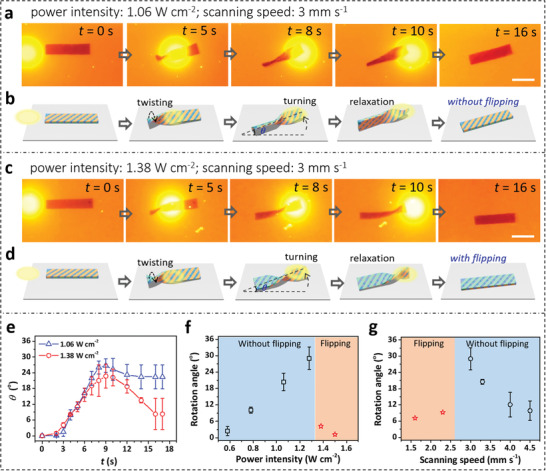
a,c) Dynamic twisting and turning motions and b,d) proposed kinematics of AuNP‐containing hydrogel strip under spatiotemporal light irradiation with different power intensity. Gel dimensions: 20 mm × 5 mm × 1 mm; light wavelength: 520 nm; scanning speed: 3 mm s^−1^. There is no flipping of the gel strip when the power intensity is 1.06 W cm^−2^ (a,b). Flipping takes place when the power intensity is 1.38 W cm^−2^ (c,d). Scale bar, 1 cm. e) Variations of instantaneous angle, *θ*, as a function of scanning time, *t*, of the light spot with different power intensities. Effects of f) power intensity and g) scanning speed on the rotation angle and flipping motion of the gel. Scanning speed is 3 mm s^−1^ in (f), and power intensity is 1.28 W cm^−2^ in (g). The photos are taken by a digital camera equipped with a cut‐off filter. Error bars represent the standard deviation of the mean (*n* = 3).

The programmed shape change of the biomimetic hydrogels can be harnessed for the design of soft actuators and robots. Here, we use light as the stimulation for site‐specific deformation of the hydrogel due to the remote and spatiotemporal control with a high resolution. This behavior is enabled by incorporating AuNPs into the gel matrix as the photothermal transducers.^[^ ^62^, ^63^ ^]^ The resultant composite gel shows good photoresponse under 520 nm light irradiation; the temperature of the gel increases to a value above the LCST of PNIPAm in 3 s with the light irradiation and recovers to room temperature in 15 s without the light irradiation (Figure S7, Supporting Information). The biomimetic gels with sophisticated anisotropic structures exhibit time‐variant shape change and programmable locomotion under spatiotemporal light irradiation. The gel strip capable of twisting is used as a proof‐of‐concept example. The hydrogel strip is actuated by dynamic scanning of a light spot having the size comparable to the width of the gel strip but much smaller than the length of the strip. As a light spot of intensity 1.06 W cm^−2^ scans left‐to‐right at a speed of 3.0 mm s^−1^, the flat gel gradually twists into a helix. The friction force generated by the curling left part of the gel strip leads to counterclockwise rotation of the gel (**Figure** **5**a,b; Movie S4, Supporting Information). The kinematics of this motion is illustrated in Figure 5b, in which the twisting moment and the center of gravity play important roles. As the light spot enters from the left side of the gel strip, the spatiotemporal plasmonic heating results in localized twisting. The friction force exerted by the substrate pushes the gel to the opposite direction, leading to the counterclockwise rotation.^[^ ^64^ ^]^ As the light spot progresses to the right of the gel strip which induces localized twisting, the left part gradually flattens due to the local cooling and relaxation of the gel matrix. After the light spot moves away from the gel strip (scanning time, *t* > 10 s), the right part also flattens without flipping. During this process, the instantaneous angle, *θ*, between the central line of the gel and the horizontal line increases from 0° to 26° as *t* elapses from 0 to 9 s, and then *θ* declines to a plateau of 22° (Figure 5e). The final plateau value is defined as the rotation/turning angle of the gel.

Interestingly, when the light intensity is increased to 1.38 W cm^−2^ (scanning speed is kept at 3.0 mm s^−1^), the twisting deformation of the gel strip is more drastic, accompanied by the flipping of the gel after a single scan of light (Figure 5c,d; Movie S5, Supporting Information). In this instance, the turning angle declines more during the relaxation and recovery process, from the maximum value of 22° to the final plateau of 8° (Figure 5e). The kinematics of the motion is illustrated in Figure 5d. When the light scans from left to right, the left part of the gel twists more due to the more intensive heating, to the extent that a large part is already flipped before the light spot goes through the center. This part remains in the flipped state during the relaxation, resulting in the flipping of the entire gel. The turning angle, on the other hand, is reduced during the relaxation, when no backward twisting takes place during relaxation.

The twisting motions with or without flipping strongly depends on the power intensity and the scanning speed of the light spot. Increasing the light intensity leads to an increase in the rotation angle of the gel, which is 30° when the light intensity is 1.28 W cm^−2^. Further increase in the light intensity results in flipping of the gel strip (Figure 5f). Accordingly, the rotation angle is reduced to a value smaller than 8° when the light intensity is higher than 1.38 W cm^−2^. Decreasing the scanning speed also results in flipping of the gel strip (Figure 5g). These results indicate that the dynamic motions of the gel are determined by the cooperative interactions of photothermal transition, localized deformation/relaxation, and dynamic reaction force. As expected, increasing the length of the gel strip results in an increase in twisting turns, which favors flipping of the gel (Figure S8, Movie S6, Supporting Information).

## 3 Conclusion

We have demonstrated a facile strategy to develop biomimetic hydrogels with intricate ordered structures of NSs that are generated by distributed electric fields from patterned electrodes, and permanently immobilized by subsequent one‐step polymerization. Through‐thickness and in‐plane gradients are encoded in the gels by tailoring the patterns and the positions of the electrodes. These composite hydrogels exhibit programmed deformations and locomotion upon heating or spatiotemporal light stimulation. The transparent ITO‐based electrodes facilitate photolithographic patterning with better control of contour profiles of the hydrogels. More sophisticated deformations and motions of the hydrogels can be developed by programming the gradient structures and using digital light stimulations.^[^ ^21^ ^]^ The dimensions of the gels can be miniaturized to micrometer level by using high‐resolution electrode patterns for the design of microactuators and robots with applications in engineering and biomedicines. The strategy of forming a distributed electric field with high versatility and controllability is potentially applicable for liquid crystals and charged molecules/particles to direct the orientation or the electrophoresis,^[^ ^42^, ^65^, ^66^ ^]^ so as to develop functional architectures and biomimetic materials with a wide‐spectrum of applications such as optical devices, flexible electronics, and soft robotics.

## 4 Experimental Section

### Materials

NIPAm was received from Aladdin Chemistry Co., Ltd.; *N*,*N′*‐methylenebis(acrylamide) (MBAA, the chemical crosslinker) was purchased from Sigma Aldrich. Lithium phenyl‐2,4,6‐trimethylbenzoylphosphinate (LAP, the photo‐initiator) was synthesized according to the method reported in the literature.^[^ ^66^ ^]^ Hectorite [Na_0.5_]^inter^[Li_0.5_Mg_2.5_]^oct^[Si_4_]^teter^O_10_F_2_ NSs were melt synthesized at high temperature followed by a long‐term annealing process.^[^ ^48^ ^]^ Simply by immersing the as‐synthesized crystals into water, utter delamination by repulsive osmotic swelling was triggered, resulting in a nematic liquid crystalline suspension. AuNPs were synthesized according to the reported method,^[^ ^67^ ^]^ and the average size was ≈10 nm.

### Hydrogel Preparation

ITO electrodes (thickness: 185 nm; light transmittance: >84%) were patterned on the glass substrate, which was assembled with another substrate with or without patterned electrodes into the reaction cell separated by 1 mm‐thick silicon spacer. The width and distance of parallel ITO stripes were 0.5 and 1 mm. Then, prescribed amount of NIPAAm (10.13 wt%), MBAA (0.276 wt%), and LAP (0.078 wt%) were dissolved in the aqueous suspension of NS (0.27 wt%) followed by addition of the aqueous suspension of AuNPs (0.23 wt%). The precursor solution was injected into the reaction cell. After applying the electric field for 60 min, NSs in the precursor solution were electrically oriented and fixed by subsequent UV irradiation (365 nm, 5 mW cm^−2^) for 50 s to complete the polymerization. The obtained hydrogel was immersed into large amount of water to remove the residuals and achieve the equilibrium state. Patterns of electrodes and dimensions of anisotropic hydrogels with programmable architectures are shown in Figures S9,S10, Supporting Information.

### Characterizations

Electrical orientation process of the NSs under the electric field and anisotropic structures of the composite hydrogels were examined by POM (LV100N POL, Nikon) and SAXS (Xeuss system) measurements. For POM observation, the hydrogel was sliced and viewed from the top and the side with and without 530 nm tint plate. For the SAXS measurement, anisotropic and isotropic regions of the gel were exposed to the X‐ray. The wavelength of X‐ray was 0.154 nm, the beam spot was 172 × 172 µm^2^, and the sample‐to‐detector distance was 1370 mm. The orientation degree (*π*) was calculated according to the equation of *π* = (180‒*H*)/180, where *H* is the half width of the peak in the intensity‐azimuthal plot from the selected equatorial reflection.

### Electric Field Simulation

The electric field calculation was carried out by using a finite element method via the commercial package COMSOL Multiphysics 5.5. The gel domain was assumed to be homogeneous and isotropic with linear electrical properties. One unit cell was modeled in each case to exploit periodicity and symmetry of the structure. Electric potentials were prescribed over the boundaries on which electrodes were attached, while symmetric or insulating conditions were assumed on all other boundaries.

### Statistical Analysis

Variations of instantaneous angle of the hydrogel strip as a function of scanning time of the light spot with different power intensities were analyzed from the snapshots of the movies that recorded the twisting and flipping process of the hydrogel (Figure 5). Each case with different power intensity and scanning speed of the light to actuate the dynamic deformation of the gel was examined by three parallel experiments. The values of instantaneous angle and rotation angle of the gel strip were presented as the mean ± standard deviation (*n* = 3).

## Conflict of Interest

The authors declare no conflict of interest.

## Supporting information

Supporting Information

Supplemental Movie 1

Supplemental Movie 2

Supplemental Movie 3

Supplemental Movie 4

Supplemental Movie 5

Supplemental Movie 6

## Data Availability

Research data are not shared.
