# Supplementary material for: Patterned Electrode Assisted One‐Step Fabrication of Biomimetic Morphing Hydrogels with Sophisticated Anisotropic Structures
Source: Adv Sci (Weinh). 2021 Oct 27;8(24):2102353. doi: 10.1002/advs.202102353 (PMC8693068; doi:10.1002/advs.202102353)
Supplement: Supplementary file 1 — Supporting Information [file ADVS-8-2102353-s006.pdf]

WILEY-VCH

© 2021 Wiley-VCH GmbH

SCIENCE
Open Access

Supporting Information

for *Adv. Sci.*, DOI: 10.1002/advs.202102353

Patterned Electrode Assisted One-Step Fabrication of Biomimetic Morphing Hydrogels with Sophisticated Anisotropic Structures

Qing Li Zhu, Chen Fei Dai, Daniel Wagner, Olena Khoruzhenko, Wei Hong, Josef Breu, Qiang Zheng, Zi Liang Wu**

Supporting Information

Patterned Electrode Assisted One-Step Fabrication of Biomimetic Morphing Hydrogels with Sophisticated Anisotropic Structures

Qing Li Zhu, Chen Fei Dai, Daniel Wagner, Olena Khoruzhenko, Wei Hong, Josef Breu, Qiang Zheng, Zi Liang Wu**

Q. L. Zhu, C. F. Dai, Prof. Q. Zheng, Prof. Z. L. Wu
Ministry of Education Key Laboratory of Macromolecular Synthesis and Functionalization
Department of Polymer Science and Engineering
Zhejiang University
Hangzhou 310027, China
E-mail: wuziliang@zju.edu.cn

D. Wagner, O. Khoruzhenko, Prof. J. Breu
Bavarian Polymer Institute and Department of Chemistry
University of Bayreuth
Universitätsstrasse 30
Bayreuth 95440, Germany
E-mail: Josef.Breu@uni-bayreuth.de

Prof. W. Hong
Department of Mechanics and Aerospace Engineering
Southern University of Science and Technology
Shenzhen 518055, China

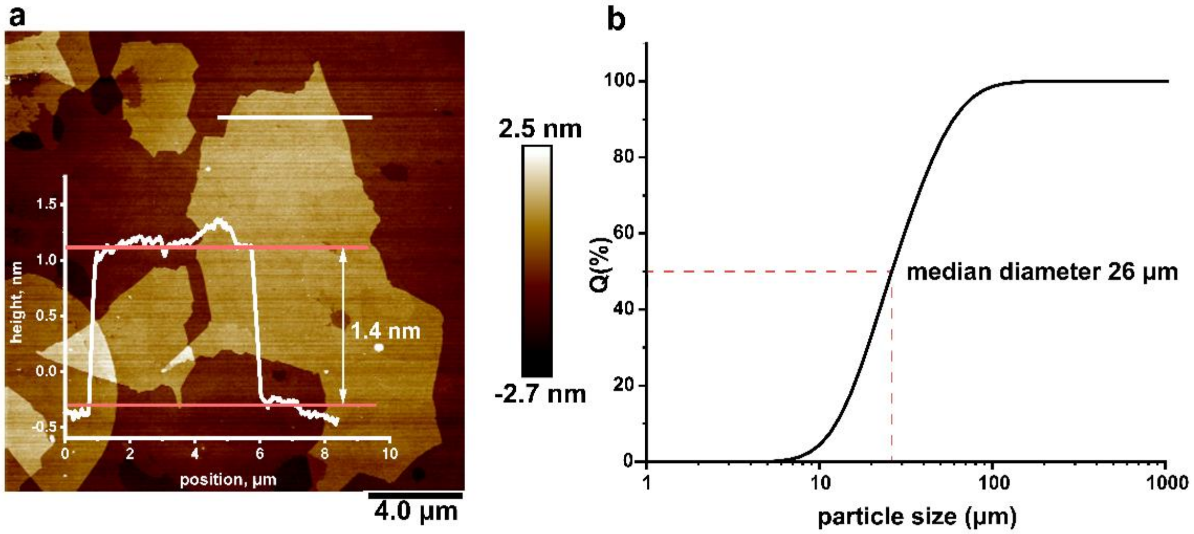

Figure S1. Characterization of the fluorohectorite nanosheets. (a) Topographic AFM image of the nanosheets and height profile (insert). (b) Particle size distribution of the nanosheets as determined by static light scattering. The median diameter is 26 μm.

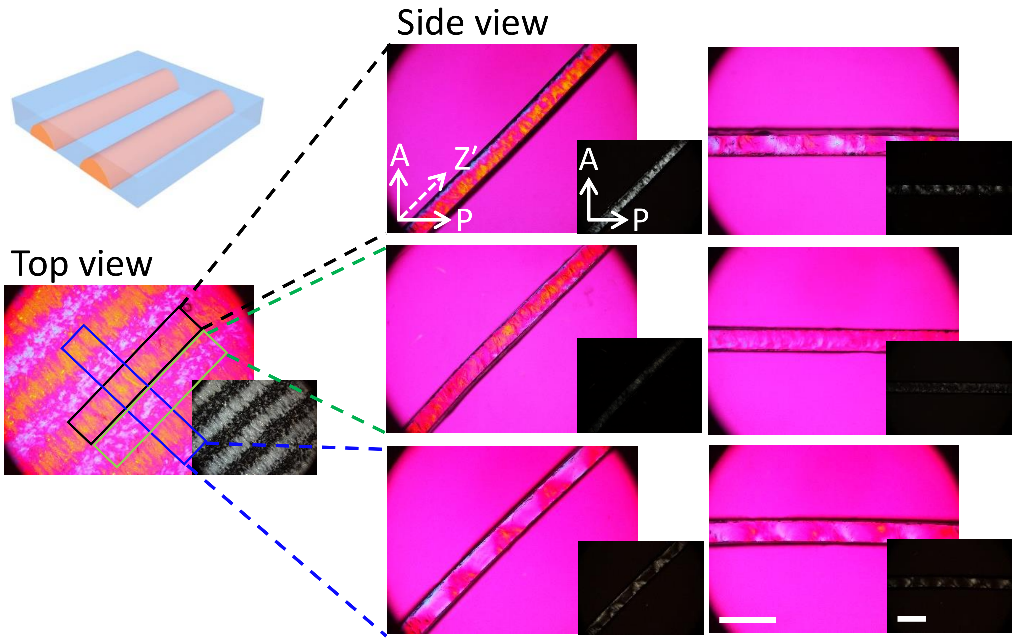

Figure S2. POM images of the hydrogel with through-thickness gradient. The hydrogel is prepared by pectinate electrodes plated on one substrate of the reaction cell (Figure 2a). The hydrogel sample is observed from the top and the side. Gel stripes are cut from the gel sheets for side-view observation. A: analyzer; P: polarizer; Z′: slow axis of the 530 nm tint plate. Scale bar, 3 mm.

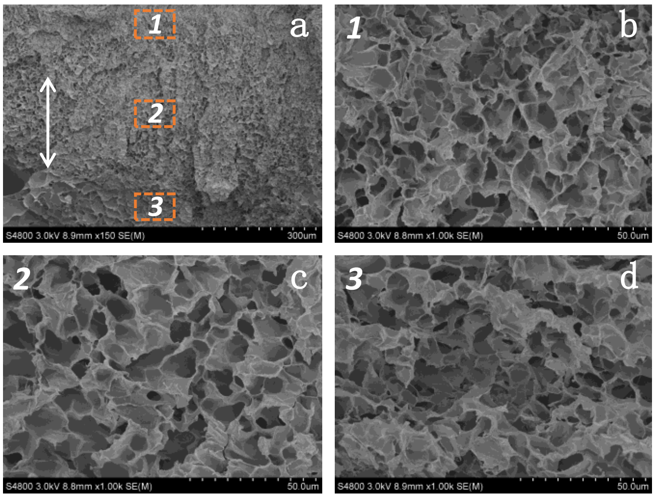

Figure S3. SEM image of the cross-section of the nanocomposite hydrogel after a freeze-drying process. White arrow in (a) indicates the thickness direction of the gel sample. Three enlarged images (b-d) are presented to show the microstructure of different places (1-3) of the gel sample, as indicated in (a).

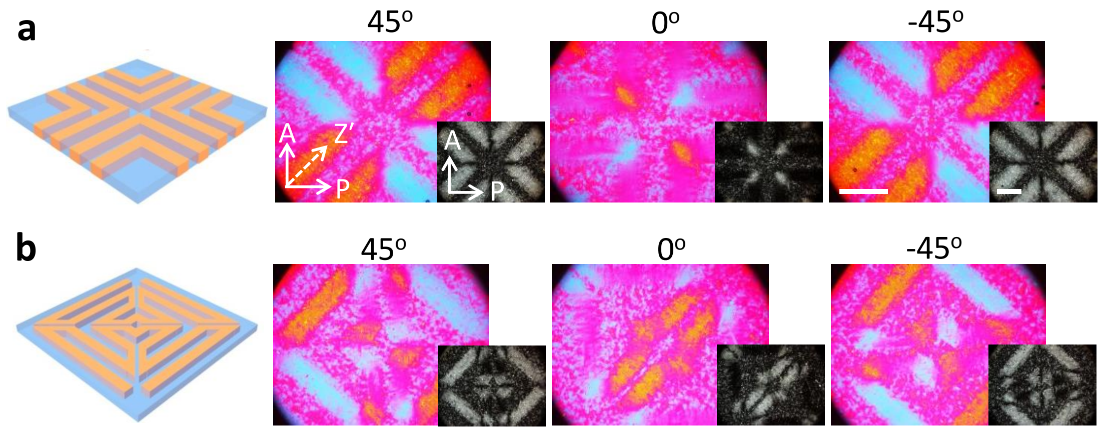

Figure S4. POM images of the hydrogels with in-plane gradients. The hydrogels are prepared two sets of pectinate electrodes plated on two substrates of the reaction cell. The patterned electrodes are assembled face-to-face to eliminate the through-thickness gradient, corresponding to Figure 3g (a) and Figure 3i (b). The samples are observed form the top and rotated by different degrees to show the birefringence patterns. Scale bar, 3 mm.

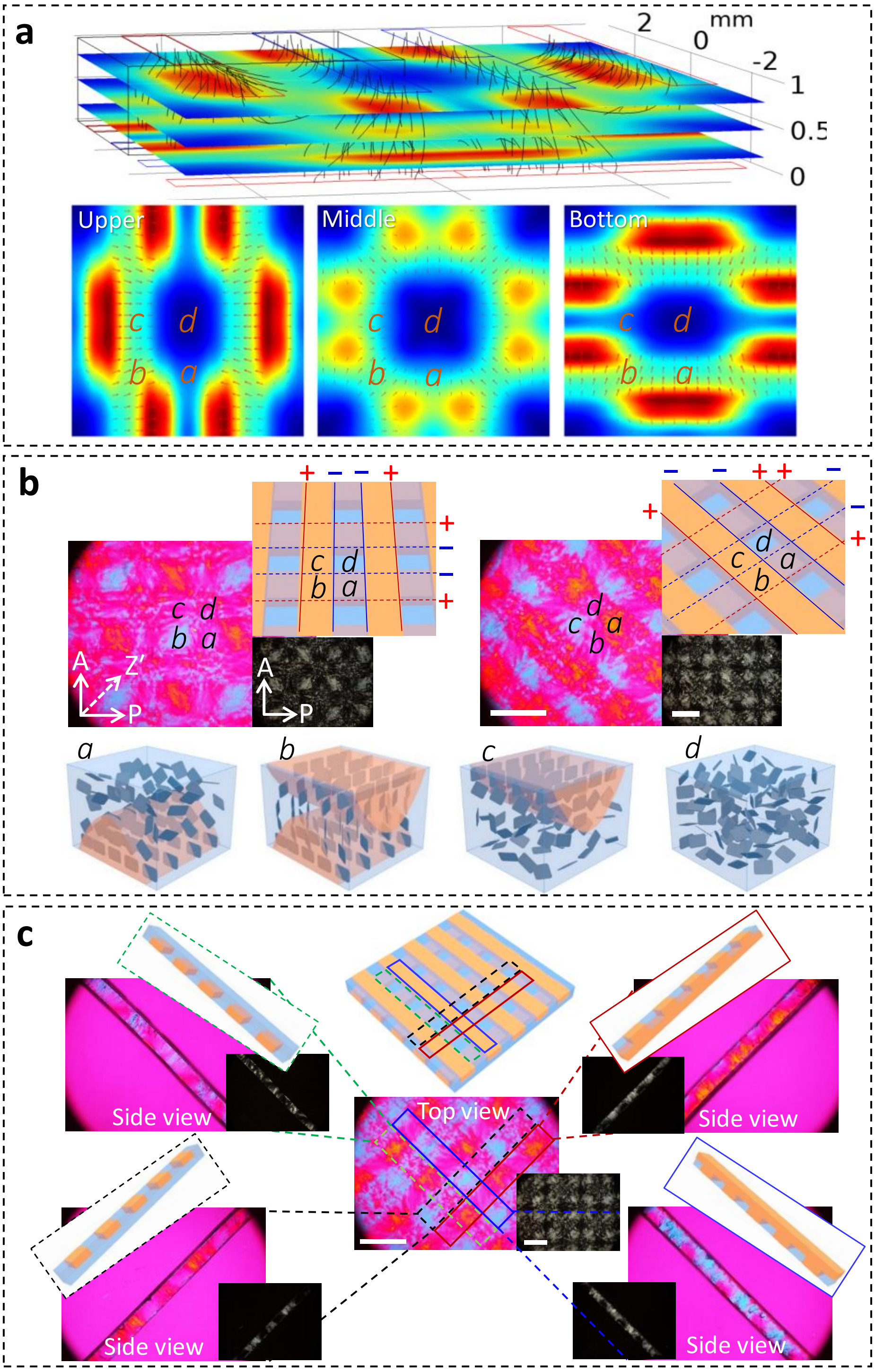

Figure S5. Characterization of sophisticated anisotropic structure of the hydrogel. (a) Simulated electric field of one unit cell when voltage is applied through the patterned electrodes. (b) POM images of the hydrogel observed from the top. Alignments of NSs at different regions are illustrated under the POM images. (c) POM images of gel stripes cut from the hydrogel sheet and observed from the side. Scale bar, 3 mm. The experimental and simulation results confirm the sophisticated alignments of NSs under the distributed electric field, as illustrated in (b). In the region *b*, the alignments of NSs are twisted by 90° from the top layer (horizontal alignment) to the bottom layer (vertical alignment); in the middle region, NSs align in northeast-southwest direction.

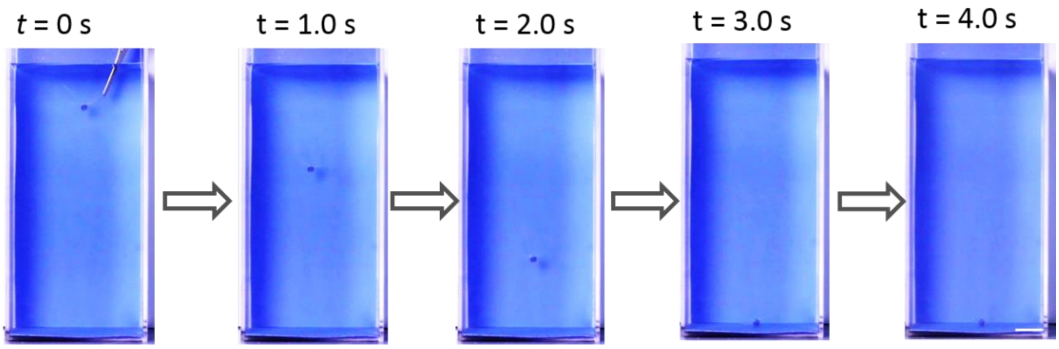

Figure S6. Free-falling process of the jellyfish-like hydrogel in 25° water. At this temperature the hydrogel keeps the original configurations with symmetric structure and falls without rotation. The gel falls from a height of 18 cm. Scale bar, 2 cm

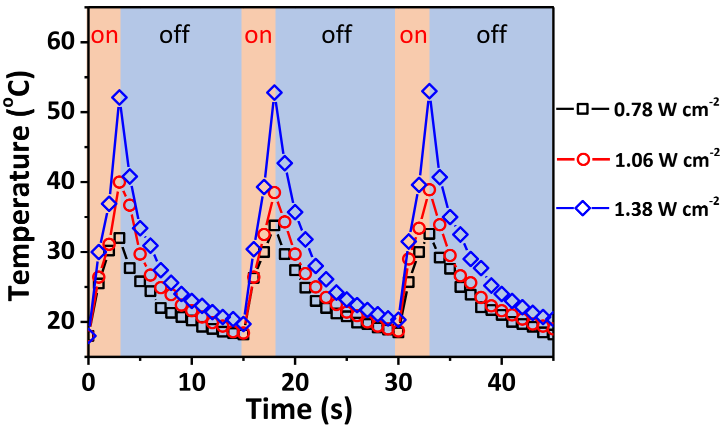

Figure S7. Photothermal effect of AuNP-containing composite hydrogel. Variations of temperature of the AuNP-containing anisotropic hydrogel upon cyclic irradiation of 520 nm light with different power intensities.

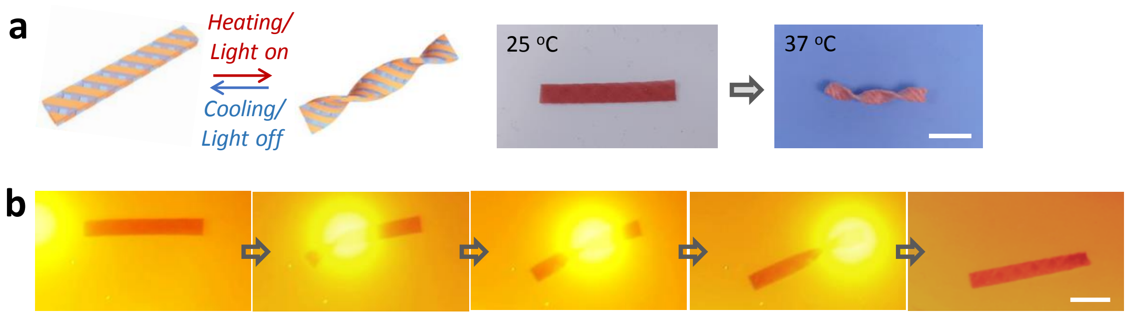

Figure S8. Morphing and motion of AuNP-containing anisotropic hydrogel. (a) Twisting deformation of the AuNP-containing hydrogel strip after transforming it from 25° to 37° water bath. (b) Twisting and flipping motions of the gel strip under scanning of 520 nm light beam from the left to the right of the gel. Gel dimensions: 30 mm × 5 mm × 1 mm; power intensity: 1.06 W cm^{-2}; scanning speed: 3 mm s^{-1}. The photos are taken by a camera equipped with a cut-off filter. Scale bar, 1 cm.

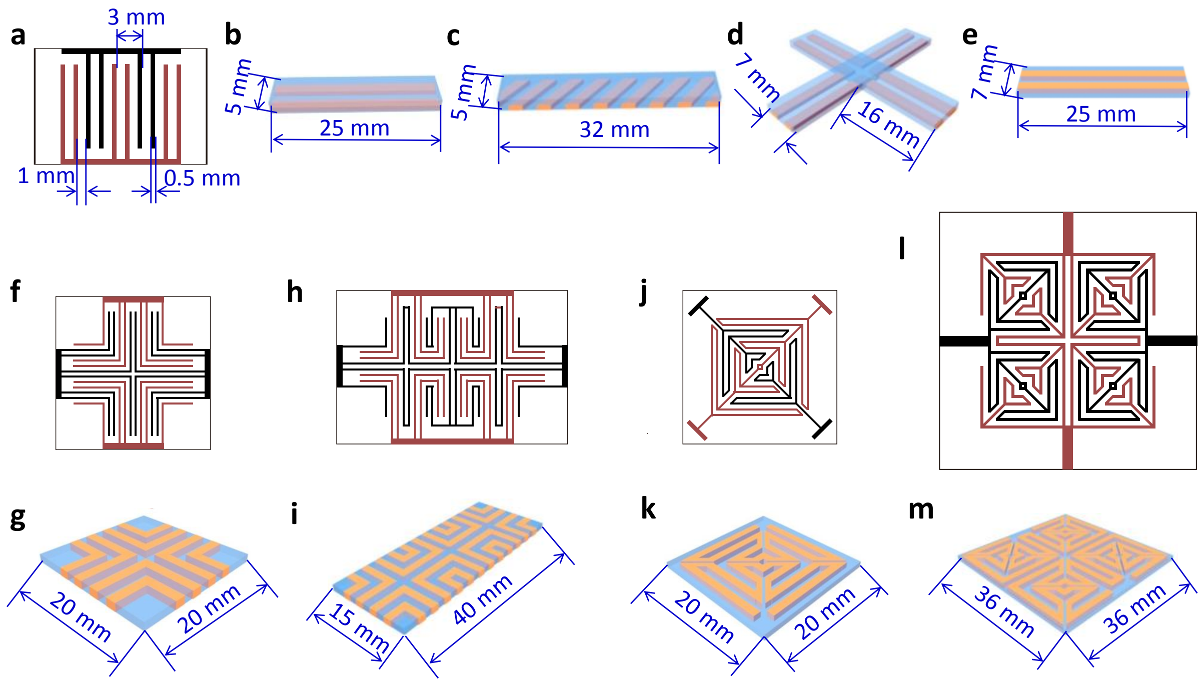

Figure S9. Patterns of electrodes and hydrogels with through-thickness or in-plane gradients. (a-d) Patterns of electrodes and dimensions of hydrogels with through-thickness gradients that are shown in Figure 2a, 3a, 4a (a), Figure 2d (b), Figure 2e (c), and Figure 2f (d). (e-m) Patterns of electrodes and dimensions of hydrogels with in-plane gradients that are shown in Figure 3e (e), Figure 3h (f and g), Figure 3i (h and i), Figure 3j (j and k), and Figure 3k (l and m). Black and red lines represent electrodes with opposite polarities. The width and the spacing of the linear electrodes in parallel arrangement are 0.5 mm and 1 mm, respectively. Orange strips represent anisotropic gels with NSs aligning perpendicular to the long axis, and blue regions represent isotropic hydrogels without long-range orientation of NSs.

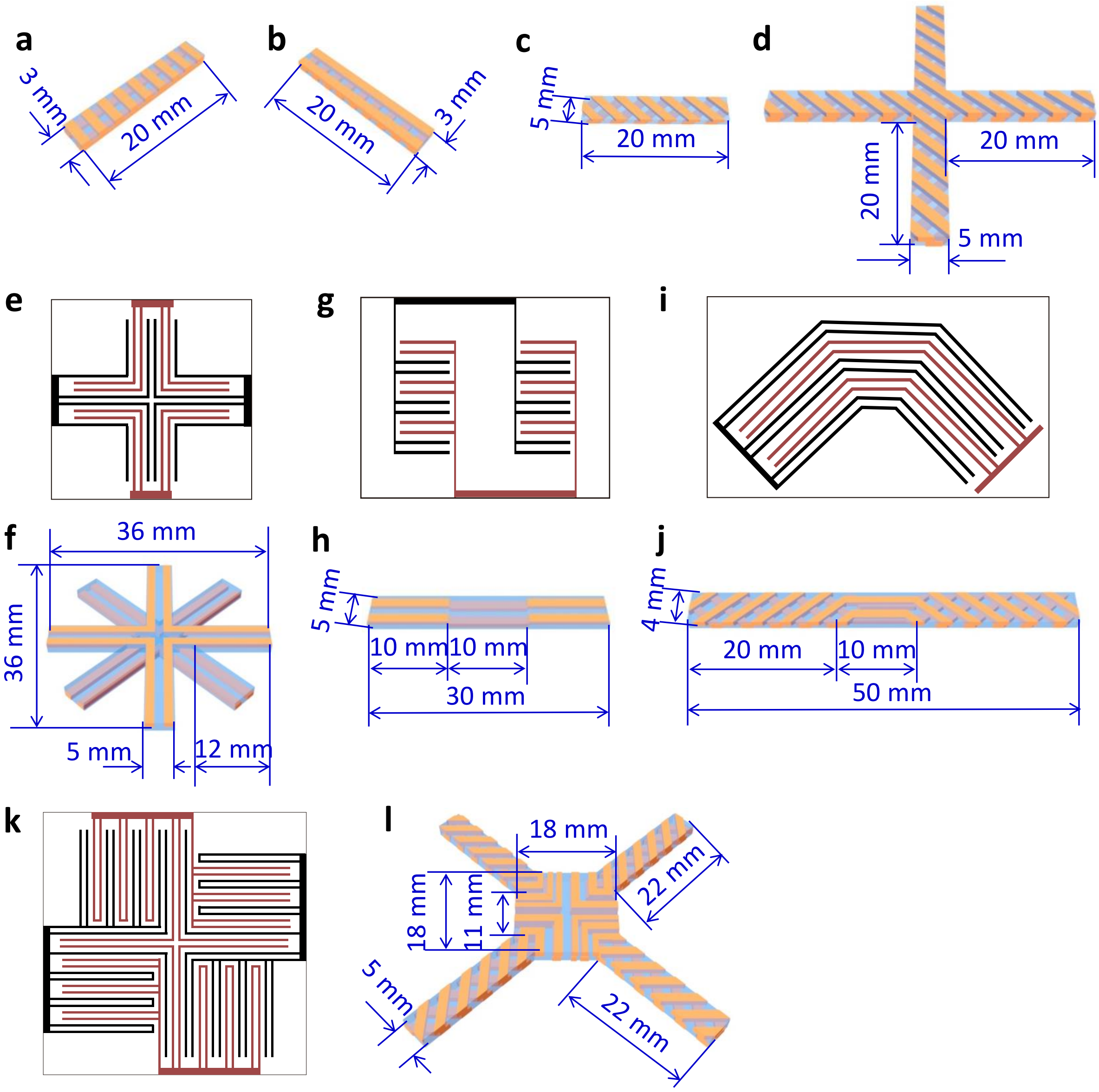

Figure S10. Patterns of electrodes and hydrogels with through-thickness and in-plane gradients. Patterns of electrodes and dimensions of hydrogels with through-thickness gradients that are shown in Figure 4d (a), Figure 4e (b), Figure 4f (c), Figure 4g (d), Figure 4h (e,f), Figure 4i (g,h), Figure 4j (i and j), and Figure 4k (k,l). The width and the spacing of the linear electrodes in parallel arrangement are 0.5 mm and 1 mm, respectively.

Captions for supplementary movies

Movie S1. Shape changing of the anisotropic hydrogel after being placed into 37 ^{o}C water bath. Movie speed, 1×.

Movie S2. Free-falling with spinning of the jellyfish-like hydrogel with chiral tentacles in 37 ^{o}C water. Movie speed, 1×.

Movie S3. Free-falling without spinning of the jellyfish-like hydrogel with achiral tentacles in 25 ^{o}C water. Movie speed, 1×.

Movie S4. Dynamic twisting (without flipping) of the gel strip under scanning of a light beam with relatively low power intensity. The movie is taken by a camera equipped with a cut-off filter. Gel dimensions: 20 mm × 5 mm × 1 mm; light intensity: 1.08 W cm^{-2}; scanning speed: 3 mm s^{-1}. Movie speed, 3×.

Movie S5. Dynamic twisting and flipping of the gel strip under scanning of a light beam with relatively high power intensity. Gel dimensions: 20 mm × 5 mm × 1 mm; light intensity: 1.38 W cm^{-2}; scanning speed: 3 mm s^{-1}. Movie speed, 3×.

Movie S6. Dynamic twisting and flipping of the gel strip with relatively longer length under scanning of a light beam. Gel dimensions: 30 mm × 5 mm × 1 mm; light intensity: 1.08 W cm^{-2}; scanning speed: 3 mm s^{-1}. Movie speed, 3×.
